# (4-Ethyl­cyclo­hex­yl)(4-meth­oxy­phen­yl)methanone

**DOI:** 10.1107/S1600536813003279

**Published:** 2013-02-06

**Authors:** Chun-Lei Ding, Yong-Jie Wu, Yi Zhang, Li Zhou

**Affiliations:** a Pharmacy Department of the Second Artillery General Hospital, Beijing 100088, People’s Republic of China; b Medical Material Purchasing Center of PLA General Hospital, Beijing 100088, People’s Republic of China

## Abstract

In the title compound, C_16_H_22_O_2_, the cyclo­hexane ring adopts a chair conformation and its mean plane subtends a dihedral angle of 54.2 (6)° with the benzene ring. The crystal structure is stabilized by van der Waals inter­actions only with no classical inter­molecular hydrogen bonding observed.

## Related literature

For details of SGLT2 inhibitors, a new class of hypoglycemic agents, see: Washburn (2009 ▶); Zhao *et al.* (2011 ▶); Shao *et al.* (2011 ▶). For the crystal structures of cyclo­hexyl derivertives, see: Wang *et al.* (2011 ▶).
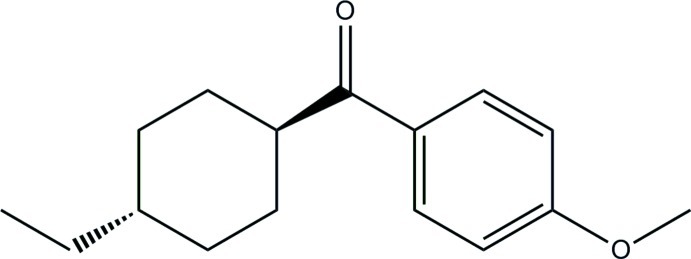


## Experimental

### 

#### Crystal data

C_16_H_22_O_2_
*M* *_r_* = 246.34
Monoclinic, 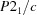

*a* = 7.613 (2) Å
*b* = 5.7513 (15) Å
*c* = 31.085 (9) Å
β = 94.674 (4)°
*V* = 1356.5 (6) Å^3^
*Z* = 4
Mo *K*α radiation
μ = 0.08 mm^−1^
*T* = 113 K
0.20 × 0.18 × 0.10 mm

#### Data collection

Rigaku Saturn CCD area-detector diffractometer
Absorption correction: multi-scan (*CrystalClear*; Rigaku/MSC, 2009) ▶ *T*_min_ = 0.985, *T*_max_ = 0.992
13145 measured reflections
3235 independent reflections
2576 reflections with *I* > 2σ(*I*)
*R*_int_ = 0.042

#### Refinement

*R*[*F* ^2^ > 2σ(*F* ^2^)] = 0.050
*wR*(*F* ^2^) = 0.139
*S* = 1.06
3235 reflections
165 parameters
H-atom parameters constrained
Δρ_max_ = 0.25 e Å^−3^
Δρ_min_ = −0.18 e Å^−3^

### 

Data collection: *CrystalClear-SM Expert* (Rigaku/MSC, 2009) ▶; cell refinement: *CrystalClear-SM Expert*; data reduction: *CrystalClear-SM Expert*; program(s) used to solve structure: *SHELXS97* (Sheldrick, 2008 ▶); program(s) used to refine structure: *SHELXL97* (Sheldrick, 2008 ▶); molecular graphics: *SHELXTL* (Sheldrick, 2008 ▶); software used to prepare material for publication: *SHELXTL*.

## Supplementary Material

Crystal structure: contains datablock(s) I, global. DOI: 10.1107/S1600536813003279/hg5288sup1.cif

Structure factors: contains datablock(s) I. DOI: 10.1107/S1600536813003279/hg5288Isup2.hkl

Supplementary material file. DOI: 10.1107/S1600536813003279/hg5288Isup3.cml

Additional supplementary materials:  crystallographic information; 3D view; checkCIF report

## Figures and Tables

**Fig. 1.**
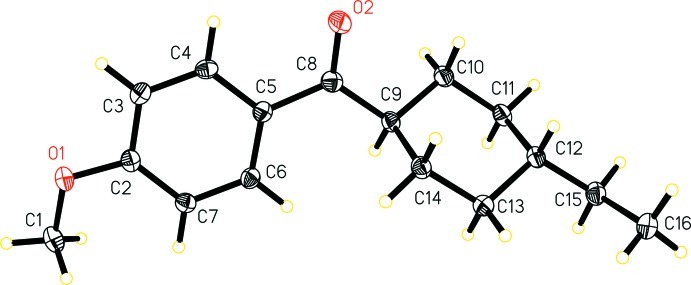
View of the title compound showing the atomic numbering and 40% probability displacement ellipsoids.

## supplementary crystallographic information

### Comment

SGLT2 inhibitors are a new class of hypoglycemic agents, and the most advanced drug dapagliflozin has been approved recently in EU for the treatment of type 2 diabetes (Washburn, 2009). During the study on the SGLT2 inhibitors in our laboratory, the title compound was prepared which is a key intermediate for the synthetic procedure (Zhao *et al.*, 2011; Shao *et al.*, 2011).

In the title compound, C_16_H_22_O_2_, bond lengths are normal and and in good agreement with those reported previously (Wang *et al.*, 2011). The cyclohexane ring adopts a chair conformation and its least squares plane (C10/C11/C13/C14) is at an angle of 54.2 (6)° to the benzene ring (C2—C7). The crystal structure is stabilized by van der Waals interactions only with no classical inter-molecular hydrogen bonding observed.

### Experimental

15.62 g (0.1 mol) of *trans*-4-ethylcyclohexanecarboxylic acid was stirred in 150 ml of dried CH_2_Cl_2_ at room temperature. 17.80 g (0.13 mol) of freshly distilled oxalyl chloride was added dropwise followed by addition of 0.1 ml of dried DMF. The mixture was stirred at room temperature for 5 h and evaporated *in vacuo* to give a residue. The residue was dissolved in 80 ml of dried dichloromethane followed by addition of 10.81 g (0.1 mol) of anisole. The mixture thus obtained was stirred at 0^o^C followed by portionwise addition of 14.67 g (0.11 mol) of AlCl_3_. The reaction mixture was then stirred at room temperature overnight, poured into 300 ml of ice-water and extracted with 100 ml three times of dichloromethane. The combined extracts were washed with brine, dried over Na_2_SO_4_ and evaporated to dryness. The residue was purified by column chromatography to afford the pure title compound as colorless crystals. The single crystals suitable for single-crystal X-ray diffraction were obtained by slow evaporation at room temperature of a 0.2 *M* solution of the title compound in dichloromethane/hexane (1/12 by *v*/*v*).

### Refinement

All H atoms bonded on carbon were found on difference maps, with C–H = 0.95–1.00, and included in the final cycles of refinement using a riding model, with *U*_iso_(H) = 1.2*U*_eq_(C) and 1.5*U*_eq_(C) for the methyl H atoms.

### Crystal data

**Table:**

C_16_H_22_O_2_	*F*(000) = 536
*M**_r_* = 246.34	*D*_x_ = 1.206 Mg m^−^^3^
Monoclinic, *P*2_1_/*c*	Mo *K*α radiation, λ = 0.71073 Å
Hall symbol: -P 2ybc	Cell parameters from 3595 reflections
*a* = 7.613 (2) Å	θ = 1.3–27.9°
*b* = 5.7513 (15) Å	µ = 0.08 mm^−^^1^
*c* = 31.085 (9) Å	*T* = 113 K
β = 94.674 (4)°	Prism, colorless
*V* = 1356.5 (6) Å^3^	0.20 × 0.18 × 0.10 mm
*Z* = 4	

### Data collection

**Table:**

Rigaku Saturn CCD area-detector diffractometer	3235 independent reflections
Radiation source: rotating anode	2576 reflections with *I* > 2σ(*I*)
Multilayer monochromator	*R*_int_ = 0.042
Detector resolution: 14.63 pixels mm^-1^	θ_max_ = 27.9°, θ_min_ = 1.3°
ω and φ scans	*h* = −9→10
Absorption correction: multi-scan (*CrystalClear*; Rigaku/MSC, 2009)	*k* = −6→7
*T*_min_ = 0.985, *T*_max_ = 0.992	*l* = −40→40
13145 measured reflections	

### Refinement

**Table:**

Refinement on *F*^2^	Primary atom site location: structure-invariant direct methods
Least-squares matrix: full	Secondary atom site location: difference Fourier map
*R*[*F*^2^ > 2σ(*F*^2^)] = 0.050	Hydrogen site location: inferred from neighbouring sites
*wR*(*F*^2^) = 0.139	H-atom parameters constrained
*S* = 1.06	*w* = 1/[σ^2^(*F*_o_^2^) + (0.0684*P*)^2^ + 0.0117*P*] where *P* = (*F*_o_^2^ + 2*F*_c_^2^)/3
3235 reflections	(Δ/σ)_max_ = 0.001
165 parameters	Δρ_max_ = 0.25 e Å^−^^3^
0 restraints	Δρ_min_ = −0.18 e Å^−^^3^

### Special details

**Table:**

Geometry. All e.s.d.'s (except the e.s.d. in the dihedral angle between two l.s. planes) are estimated using the full covariance matrix. The cell e.s.d.'s are taken into account individually in the estimation of e.s.d.'s in distances, angles and torsion angles; correlations between e.s.d.'s in cell parameters are only used when they are defined by crystal symmetry. An approximate (isotropic) treatment of cell e.s.d.'s is used for estimating e.s.d.'s involving l.s. planes.
Refinement. Refinement of *F*^2^ against ALL reflections. The weighted *R*-factor *wR* and goodness of fit *S* are based on *F*^2^, conventional *R*-factors *R* are based on *F*, with *F* set to zero for negative *F*^2^. The threshold expression of *F*^2^ > σ(*F*^2^) is used only for calculating *R*-factors(gt) *etc*. and is not relevant to the choice of reflections for refinement. *R*-factors based on *F*^2^ are statistically about twice as large as those based on *F*, and *R*- factors based on ALL data will be even larger.

### Fractional atomic coordinates and isotropic or equivalent isotropic displacement parameters (Å^2^)

**Table:**

	*x*	*y*	*z*	*U*_iso_*/*U*_eq_	
O1	0.34006 (12)	0.74119 (17)	0.16856 (3)	0.0272 (3)	
O2	0.05937 (15)	0.53751 (18)	0.34949 (3)	0.0352 (3)	
C1	0.3969 (2)	0.9518 (3)	0.14967 (4)	0.0316 (4)	
H1A	0.3107	1.0750	0.1536	0.047*	
H1B	0.4073	0.9276	0.1188	0.047*	
H1C	0.5117	0.9974	0.1637	0.047*	
C2	0.29562 (17)	0.7517 (2)	0.21018 (4)	0.0215 (3)	
C3	0.20950 (17)	0.5560 (2)	0.22446 (4)	0.0229 (3)	
H3	0.1869	0.4273	0.2057	0.028*	
C4	0.15706 (17)	0.5488 (2)	0.26592 (4)	0.0212 (3)	
H4	0.0986	0.4147	0.2755	0.025*	
C5	0.18896 (17)	0.7370 (2)	0.29413 (4)	0.0198 (3)	
C6	0.27745 (17)	0.9302 (2)	0.27951 (4)	0.0215 (3)	
H6	0.3013	1.0582	0.2984	0.026*	
C7	0.33165 (18)	0.9393 (2)	0.23779 (4)	0.0215 (3)	
H7	0.3923	1.0718	0.2283	0.026*	
C8	0.12147 (18)	0.7220 (2)	0.33787 (4)	0.0229 (3)	
C9	0.13144 (17)	0.9305 (2)	0.36774 (4)	0.0213 (3)	
H9	0.1151	1.0748	0.3499	0.026*	
C10	−0.01352 (18)	0.9211 (2)	0.39925 (4)	0.0245 (3)	
H10A	−0.1303	0.9292	0.3828	0.029*	
H10B	−0.0061	0.7714	0.4150	0.029*	
C11	0.00388 (17)	1.1215 (2)	0.43170 (4)	0.0239 (3)	
H11A	−0.0890	1.1069	0.4521	0.029*	
H11B	−0.0144	1.2708	0.4161	0.029*	
C12	0.18477 (18)	1.1241 (2)	0.45716 (4)	0.0219 (3)	
H12	0.2001	0.9730	0.4730	0.026*	
C13	0.32707 (18)	1.1398 (2)	0.42528 (4)	0.0235 (3)	
H13A	0.4446	1.1378	0.4414	0.028*	
H13B	0.3147	1.2892	0.4095	0.028*	
C14	0.31498 (18)	0.9391 (2)	0.39280 (4)	0.0233 (3)	
H14A	0.3376	0.7903	0.4083	0.028*	
H14B	0.4064	0.9588	0.3722	0.028*	
C15	0.19885 (19)	1.3203 (3)	0.49047 (4)	0.0264 (3)	
H15A	0.2086	1.4700	0.4751	0.032*	
H15B	0.0886	1.3248	0.5053	0.032*	
C16	0.3545 (2)	1.2989 (3)	0.52446 (4)	0.0319 (4)	
H16A	0.3385	1.1616	0.5424	0.048*	
H16B	0.3609	1.4383	0.5427	0.048*	
H16C	0.4639	1.2832	0.5101	0.048*	

### Atomic displacement parameters (Å^2^)

**Table:**

	*U*^11^	*U*^22^	*U*^33^	*U*^12^	*U*^13^	*U*^23^
O1	0.0309 (6)	0.0321 (6)	0.0196 (5)	−0.0005 (4)	0.0068 (4)	−0.0025 (4)
O2	0.0550 (7)	0.0257 (6)	0.0261 (6)	−0.0095 (5)	0.0099 (5)	0.0028 (4)
C1	0.0357 (9)	0.0380 (10)	0.0223 (7)	0.0010 (7)	0.0089 (6)	0.0029 (6)
C2	0.0185 (7)	0.0272 (8)	0.0186 (6)	0.0026 (6)	0.0004 (5)	−0.0004 (5)
C3	0.0230 (7)	0.0221 (8)	0.0234 (7)	0.0009 (6)	0.0000 (5)	−0.0032 (5)
C4	0.0197 (7)	0.0188 (7)	0.0247 (7)	−0.0007 (5)	−0.0008 (5)	0.0022 (5)
C5	0.0200 (7)	0.0209 (7)	0.0180 (6)	0.0014 (5)	−0.0012 (5)	0.0012 (5)
C6	0.0231 (7)	0.0220 (8)	0.0188 (7)	0.0000 (6)	−0.0010 (5)	−0.0005 (5)
C7	0.0212 (7)	0.0216 (8)	0.0218 (7)	−0.0007 (5)	0.0018 (5)	0.0016 (5)
C8	0.0233 (7)	0.0232 (8)	0.0216 (7)	0.0001 (6)	−0.0008 (5)	0.0038 (5)
C9	0.0252 (8)	0.0221 (8)	0.0167 (6)	−0.0005 (6)	0.0022 (5)	0.0025 (5)
C10	0.0237 (8)	0.0296 (8)	0.0203 (7)	−0.0025 (6)	0.0021 (5)	0.0020 (5)
C11	0.0224 (7)	0.0300 (8)	0.0198 (6)	0.0019 (6)	0.0057 (5)	0.0019 (5)
C12	0.0251 (7)	0.0233 (8)	0.0177 (6)	0.0006 (6)	0.0034 (5)	0.0015 (5)
C13	0.0229 (7)	0.0257 (8)	0.0221 (7)	−0.0018 (6)	0.0026 (5)	0.0001 (5)
C14	0.0230 (7)	0.0267 (8)	0.0207 (7)	0.0005 (6)	0.0043 (5)	−0.0007 (5)
C15	0.0316 (8)	0.0260 (8)	0.0221 (7)	0.0012 (6)	0.0043 (6)	−0.0015 (5)
C16	0.0367 (9)	0.0360 (9)	0.0230 (7)	−0.0035 (7)	0.0025 (6)	−0.0045 (6)

### Geometric parameters (Å, º)

**Table:**

O1—C2	1.3649 (15)	C10—C11	1.5302 (18)
O1—C1	1.4286 (16)	C10—H10A	0.9900
O2—C8	1.2281 (16)	C10—H10B	0.9900
C1—H1A	0.9800	C11—C12	1.5313 (18)
C1—H1B	0.9800	C11—H11A	0.9900
C1—H1C	0.9800	C11—H11B	0.9900
C2—C7	1.3923 (18)	C12—C13	1.5294 (18)
C2—C3	1.3933 (19)	C12—C15	1.5295 (18)
C3—C4	1.3805 (18)	C12—H12	1.0000
C3—H3	0.9500	C13—C14	1.5315 (18)
C4—C5	1.4017 (18)	C13—H13A	0.9900
C4—H4	0.9500	C13—H13B	0.9900
C5—C6	1.3941 (18)	C14—H14A	0.9900
C5—C8	1.4946 (18)	C14—H14B	0.9900
C6—C7	1.3935 (17)	C15—C16	1.5269 (19)
C6—H6	0.9500	C15—H15A	0.9900
C7—H7	0.9500	C15—H15B	0.9900
C8—C9	1.5146 (18)	C16—H16A	0.9800
C9—C10	1.5352 (18)	C16—H16B	0.9800
C9—C14	1.5441 (18)	C16—H16C	0.9800
C9—H9	1.0000		
			
C2—O1—C1	117.31 (11)	C9—C10—H10B	109.3
O1—C1—H1A	109.5	H10A—C10—H10B	108.0
O1—C1—H1B	109.5	C10—C11—C12	111.94 (11)
H1A—C1—H1B	109.5	C10—C11—H11A	109.2
O1—C1—H1C	109.5	C12—C11—H11A	109.2
H1A—C1—H1C	109.5	C10—C11—H11B	109.2
H1B—C1—H1C	109.5	C12—C11—H11B	109.2
O1—C2—C7	124.55 (12)	H11A—C11—H11B	107.9
O1—C2—C3	115.20 (12)	C13—C12—C15	112.37 (11)
C7—C2—C3	120.25 (12)	C13—C12—C11	108.69 (11)
C4—C3—C2	120.06 (12)	C15—C12—C11	111.55 (11)
C4—C3—H3	120.0	C13—C12—H12	108.0
C2—C3—H3	120.0	C15—C12—H12	108.0
C3—C4—C5	120.83 (13)	C11—C12—H12	108.0
C3—C4—H4	119.6	C12—C13—C14	112.00 (11)
C5—C4—H4	119.6	C12—C13—H13A	109.2
C6—C5—C4	118.36 (12)	C14—C13—H13A	109.2
C6—C5—C8	123.61 (12)	C12—C13—H13B	109.2
C4—C5—C8	118.01 (12)	C14—C13—H13B	109.2
C7—C6—C5	121.40 (12)	H13A—C13—H13B	107.9
C7—C6—H6	119.3	C13—C14—C9	111.10 (11)
C5—C6—H6	119.3	C13—C14—H14A	109.4
C2—C7—C6	119.08 (12)	C9—C14—H14A	109.4
C2—C7—H7	120.5	C13—C14—H14B	109.4
C6—C7—H7	120.5	C9—C14—H14B	109.4
O2—C8—C5	119.12 (12)	H14A—C14—H14B	108.0
O2—C8—C9	120.34 (12)	C16—C15—C12	114.64 (12)
C5—C8—C9	120.54 (12)	C16—C15—H15A	108.6
C8—C9—C10	111.14 (11)	C12—C15—H15A	108.6
C8—C9—C14	109.43 (11)	C16—C15—H15B	108.6
C10—C9—C14	110.31 (11)	C12—C15—H15B	108.6
C8—C9—H9	108.6	H15A—C15—H15B	107.6
C10—C9—H9	108.6	C15—C16—H16A	109.5
C14—C9—H9	108.6	C15—C16—H16B	109.5
C11—C10—C9	111.47 (11)	H16A—C16—H16B	109.5
C11—C10—H10A	109.3	C15—C16—H16C	109.5
C9—C10—H10A	109.3	H16A—C16—H16C	109.5
C11—C10—H10B	109.3	H16B—C16—H16C	109.5
			
C1—O1—C2—C7	−12.28 (19)	O2—C8—C9—C10	−27.46 (17)
C1—O1—C2—C3	168.14 (11)	C5—C8—C9—C10	153.29 (12)
O1—C2—C3—C4	−179.40 (11)	O2—C8—C9—C14	94.61 (15)
C7—C2—C3—C4	1.0 (2)	C5—C8—C9—C14	−84.64 (14)
C2—C3—C4—C5	0.1 (2)	C8—C9—C10—C11	175.81 (10)
C3—C4—C5—C6	−1.00 (19)	C14—C9—C10—C11	54.25 (14)
C3—C4—C5—C8	177.29 (12)	C9—C10—C11—C12	−56.99 (14)
C4—C5—C6—C7	0.78 (19)	C10—C11—C12—C13	57.33 (14)
C8—C5—C6—C7	−177.40 (12)	C10—C11—C12—C15	−178.23 (11)
O1—C2—C7—C6	179.23 (12)	C15—C12—C13—C14	178.48 (10)
C3—C2—C7—C6	−1.21 (19)	C11—C12—C13—C14	−57.57 (14)
C5—C6—C7—C2	0.3 (2)	C12—C13—C14—C9	57.13 (15)
C6—C5—C8—O2	−173.42 (12)	C8—C9—C14—C13	−176.81 (11)
C4—C5—C8—O2	8.38 (19)	C10—C9—C14—C13	−54.24 (14)
C6—C5—C8—C9	5.83 (19)	C13—C12—C15—C16	−72.27 (15)
C4—C5—C8—C9	−172.36 (12)	C11—C12—C15—C16	165.39 (12)
